# Bromodomain inhibitor OTX015 (MK-8628) combined with targeted agents shows strong *in vivo* antitumor activity in lymphoma

**DOI:** 10.18632/oncotarget.10983

**Published:** 2016-08-01

**Authors:** Eugenio Gaudio, Chiara Tarantelli, Maurilio Ponzoni, Elodie Odore, Keyvan Rezai, Elena Bernasconi, Luciano Cascione, Andrea Rinaldi, Anastasios Stathis, Eugenia Riveiro, Esteban Cvitkovic, Emanuele Zucca, Francesco Bertoni

**Affiliations:** ^1^ Lymphoma and Genomics Research Program, Institute of Oncology Research (IOR), Bellinzona, Switzerland; ^2^ San Raffaele Scientific Institute, Milan, Italy; ^3^ Institut Curie, Hôpital René Huguenin, Saint-Cloud, France; ^4^ Oncology Institute of Southern Switzerland (IOSI), Bellinzona, Switzerland; ^5^ Oncology Therapeutic Development, Clichy, France

**Keywords:** BET inhibitor, ibrutinib, rituximab, vorinostat, everolimus

## Abstract

The bromodomain inhibitor OTX015 (MK-8628) has shown anti-lymphoma activity as a single agent in both the preclinical and clinical settings, as well as *in vitro* synergism with several anticancer agents. Here, we report *in vivo* data for OTX015 in combination with the histone deacetylase inhibitor vorinostat, the Bruton's tyrosine kinase inhibitor ibrutinib, the anti-CD20 monoclonal antibody rituximab, and the mTOR inhibitor everolimus in a diffuse large B cell lymphoma model. The antitumor effect of OTX015-containing combinations in SU-DHL-2 xenografts in mice was much stronger than the activity of the corresponding single agents with almost complete tumor eradication for all four combinations. Pharmacokinetic analyses showed similar OTX015 levels in plasma and tumor samples of approximately 1.5 μM, which is equivalent to the concentration showing strong *in vitro* activity. For all four combinations, mean terminal levels of the bromodomain inhibitor differed from those in mice exposed to single agent OTX015, indicating a need for thorough pharmacokinetic investigations in phase I combination studies. In conclusion, our results provide a strong rationale to explore OTX015-containing combinations in the clinical lymphoma setting.

## INTRODUCTION

Diffuse large B cell lymphoma (DLBCL) is the most common lymphoma in Western countries and, despite improvements obtained with chemoimmunotherapy, up to half of these patients cannot be cured [1, 2]. Currently, two main DLBCL subtypes are recognized based on their phenotypic homology with their putative cell of origin, the germinal center B-cell type and the activated B-cell like (ABC) type [2–5]. ABC-DLBCL is less responsive to standard regimens and is characterized by activation of B-cell receptor signaling and the nuclear factor kB pathway [2–5], providing therapeutic targets that are currently being explored in the clinic with compounds such as the Bruton's tyrosine kinase (BTK) inhibitor ibrutinib [6].

Analogous to the vast majority of human tumors and independent of their cell of origin, DLBCL cells also bear recurrent somatic mutations in genes coding for proteins involved in chromatin structure and remodeling that cause profound changes at the epigenetic level [3, 7]. Of clinical relevance, epigenetic changes can be at least partially reversed and epigenetic drugs can increase sensitivity to other anticancer agents [7–10]. Over the last few years, inhibitors of the bromodomain and extraterminal (BET) protein family have become the focus of extensive research as a novel class of epigenetic drugs [11]. BET proteins are key epigenetic regulators of gene transcription and their inhibition has resulted in antitumor activity in different tumor models, including lymphomas [11–20]. OTX015 (MK-8628) is a thienotriazolodiazepine compound that potently inhibits the BET proteins BRD2, BRD3 and BRD4 [21]. The compound competitively occupies the acetyl-binding pockets of BET bromodomains, leading to release of the BET protein from the chromatin [21]. Importantly, in normal and cancer cells, more than half of all BRD4 proteins are bound to a small number of enhancers (super-enhancers) that control the expression of genes fundamental to the control and establishment of individual cell identities, such as PAX5, BCL6, CD79A, CD79B, FOXO1 in B-cells or PRDM1, IRF4 and MUM1 in plasma cells [22–24].

OTX015 has *in vitro* and *in vivo* antitumor activity as a single agent in different lymphoma models, including ABC-DLBCL [14]. Clinical responses including complete remissions with single agent OTX015 have been recently reported in patients with relapsed or refractory lymphoma or acute leukemia enrolled in phase I studies, in the absence of major toxicities [25, 26]. Although the mechanism of action of BET inhibitors is likely pleiotropic, down-regulation of genes involved in B cell identity and germinal center formation, and, especially in the ABC-DLBCL setting in which such effects can lead to apoptosis, inhibition of the B-cell receptor and nuclear factor kB signaling pathways play an important role [12–14]. Since OTX015 presented *in vitro* synergism when combined with different agents in lymphoma models [14], we evaluated the *in vivo* activity of OTX015-containing combinations in an ABC-DLCBL xenograft model.

## RESULTS AND DISCUSSION

**Figure 1:**
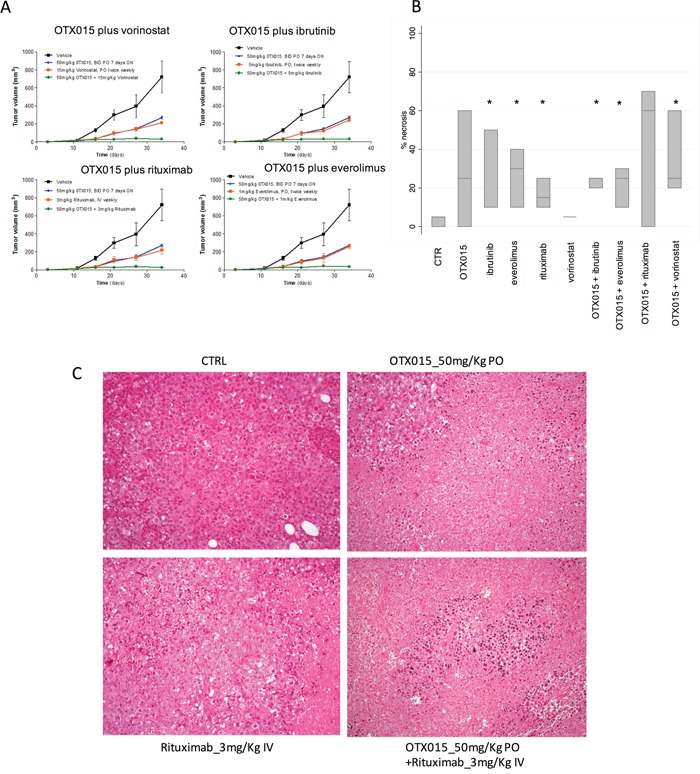
*In vivo* treatment of ABC-DLBCL SU-DHL-2 xenografts with OTX015 as a single agent and in combination with other targeted drugs **A.** Changes in tumor volumes during treatment: Black, vehicle (control mice); Blue; single agent OTX015; Red, single agent targeted drug; Green, OTX015/targeted drug combination. **B.** Boxplots showing percentage of tumor necrosis at the end of treatment. In each boxplot, the line in the middle of the box represents the median and the box extends from the 25th to the 75th percentile (interquartile range). * P < 0.05 when compared with control (CTR) mice. **C.** Histopathological analysis revealed control mice or treated only with rituximab displayed vital cell with a diffuse growth pattern (upper and lower left); addition of OTX015 was associated with large areas of coagulative necrosis (Haematoxyln and Eosin, 200X).

Based on the *in vitro* synergism observed for combinations of OTX015 with other compounds [14], we evaluated the activity of combinations of this bromodomain inhibitor in an *in vivo* model of ABC-DLBCL. Mice bearing xenografts of the ABC-DLBCL cell line SU-DHL-2 were treated with control or OTX015, BTK inhibitor ibrutinib, the mechanistic target of rapamycin (mTOR) inhibitor everolimus, the histone deacetylase inhibitor vorinostat, or the anti-CD20 monoclonal antibody rituximab as single agents or in OTX015-containing combinations. None of the mice showed any body weight loss during the treatment period. When given as single agents, OTX015 and all four other drugs caused tumor growth delay (Figure 1A). When given in combination, the antitumor activity was significantly greater, with an almost complete and immediate tumor eradication in mice receiving the OTX015-containing combinations, maintained throughout treatment (P<0.001) (Figure 1A). The degree of necrosis was also evaluated in three tumors per group. Tumors from mice treated with rituximab (P=0.0463), everolimus (P=0.0463) or ibrutinib (P=0.0431) as single agents, or with OTX015 combinations plus everolimus (P=0.0463), plus ibrutinib (P=0.0431), and plus vorinostat (P=0.0463) presented a higher percentage of necrotic cells than control mice (Figure 1B, 1C). Higher necrosis was observed in tumors from mice treated with the OTX015 and vorinostat combination compared to the single agent vorinostat group (P=0.0109). Together with our previous *in vitro* findings with OTX015 as a single agent and in combination [14], the OTX015 antitumor activity reported as single agent in the phase I hematologic study [25], and similar positive results of other combination regimens based on BET inhibitors [13, 16–18], these novel *in vivo* data confirm the combinability of OTX015 with classic cytotoxic and targeted therapies in lymphoma and provide supporting rationale for future clinical development strategies in lymphoma. Due to the direct effect of OTX015 and other BET inhibitors on MYC expression, independently of the presence of chromosomal translocations [14], also high-risk populations such the double-hit or double-expressor lymphomas [4] could be targeted.

**Figure 2:**
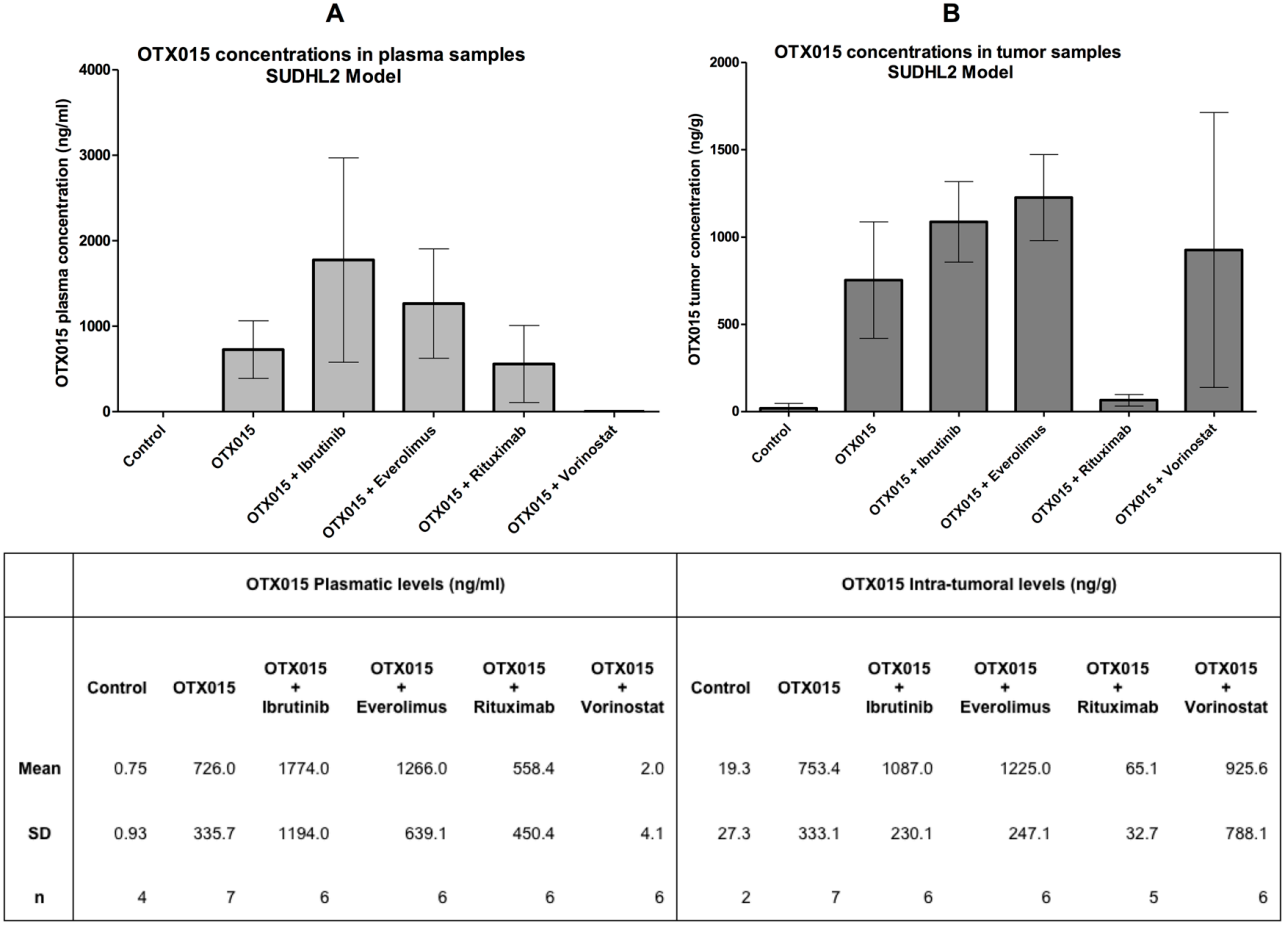
OTX015 levels in plasma and tumor tissue of SUDHL2-tumor bearing mice as a single agent or in combination with other agents **A.** Plasma and **B.** tissue levels of OTX015 (oral 50 mg/kg/day) were measured after 5 weeks treatment, 4 h after the last OTX015 administration. Each bar represents the mean ± standard deviation.

Pharmacokinetics analyses showed similar OTX015 levels in plasma and tumor samples 4 h after the last treatment when administrated as a single agent, with values of ~750 ng/ml in plasma, which is equivalent to the 1.5 μM concentration that has strong *in vitro* activity [14], and ~750 ng/g of tissue for tumor samples (Figure 2A–2B). Terminal levels of the bromodomain inhibitor in all experimental groups treated with OTX015 in combination, differed from the group exposed to OTX015 as single agent. Co-treatment with ibrutinib or everolimus induced an increase in OTX015 concentrations both in plasma (Figure 2A) and tumor samples (Figure 2B). On the other hand, treatment with rituximab decreased OTX015 accumulation in the tumor tissue and OTX015 was not detected in plasma samples in mice concomitantly receiving vorinostat. These results are based on a limited number of mice but suggest that extended pharmacokinetic/pharmacodynamic studies should be mandatory in phase I combination studies to explore the behavior of OTX015 when administered with other agents.

In conclusion, OTX015 showed strong *in vivo* activity in a murine xenograft model of ABC-DLBCL when combined with ibrutinib, everolimus, rituximab, or vorinostat. Our results provide the rationale to explore OTX015-containing combinations in the clinical setting.

## MATERIALS AND METHODS

NOD-Scid (NOD.CB17-*Prkdcscid*/NCrHsd) mice (five weeks of age, approximately 20 g body weight; Harlan Laboratory, S. Pietro al Natisone, Udine, Italy) were subcutaneously engrafted with 15 x10^6^ cells of the human ABC-DLBCL cell line SU-DHL-2, and randomly divided into 10 groups of six mice each. Treatment with OTX015 (50 mg/kg once daily, oral, qdx7/w x5w; Oncoethix GmbH, a wholly owned subsidiary of Merck Sharp & Dohme Corp, Lucerne, Switzerland; formerly Oncoethix SA) was initiated three days after the engraftment, while treatment with ibrutinib (5 mg/kg PO; qdx2/w x5w), everolimus (1 mg/kg PO; qdx2/w x5w), vorinostat (15 mg/kg PO; qdx2/w x5w; Selleckchem, Houston, TX, USA) or rituximab (3 mg/kg IV; qdx1/w x5w; Roche, Basel, Switzerland) was initiated when mice developed palpable tumors (100 mm^3^). Tumor size was measured twice weekly using a digital caliper. Tumor volumes were calculated as previously described [27]. Mice maintenance and animal experiments were performed with study protocols approved by the local Swiss Cantonal Veterinary Authority (No. 10/2014). Tumor specimens were collected at the end of treatment. Necrosis was semi-quantitatively assessed on hematoxylin-eosin stained slides. The percentage of necrotic cells on the total amount of the neoplastic tissue was evaluated on the whole section in 3 mice per each group. Differences in tumor volumes and percentage of necrosis were calculated using the Wilcoxon rank-sum test (Stata/SE 12.1 for Mac, Stata Corporation).

For OTX015 plasma and tissue concentrations, samples were collected 4 h after the last OTX015 treatment. Mice were sacrificed and blood was collected from the heart in heparinized tubes, separated immediately by centrifugation (4000 rpm, 15 min, 4°C), and stored at −80°C. Plasma concentrations were measured using a validated Ultra Performance Liquid Chromatography with tandem Mass Spectrometry method, as previously described [28]. For tissue measurements, frozen samples were weighed and then homogenized in 1 mL of water, and a 50 μL sample was prepared using the same extraction method as that used for plasma samples.
